# Suppression of non-radiative surface recombination by N incorporation in GaAs/GaNAs core/shell nanowires

**DOI:** 10.1038/srep11653

**Published:** 2015-06-23

**Authors:** Shula L. Chen, Weimin M. Chen, Fumitaro Ishikawa, Irina A. Buyanova

**Affiliations:** 1 Department of Physics, Chemistry and Biology, Linköping University, 58183, Linköping, Sweden; 2 Graduate School of Science and Engineering, Ehime University, Matsuyama 790-8577, Japan

## Abstract

III-V semiconductor nanowires (NWs) such as GaAs NWs form an interesting artificial materials system promising for applications in advanced optoelectronic and photonic devices, thanks to the advantages offered by the 1D architecture and the possibility to combine it with the main-stream silicon technology. Alloying of GaAs with nitrogen can further enhance performance and extend device functionality via band-structure and lattice engineering. However, due to a large surface-to-volume ratio, III-V NWs suffer from severe non-radiative carrier recombination at/near NWs surfaces that significantly degrades optical quality. Here we show that increasing nitrogen composition in novel GaAs/GaNAs core/shell NWs can strongly suppress the detrimental surface recombination. This conclusion is based on our experimental finding that lifetimes of photo-generated free excitons and free carriers increase with increasing N composition, as revealed from our time-resolved photoluminescence (PL) studies. This is accompanied by a sizable enhancement in the PL intensity of the GaAs/GaNAs core/shell NWs at room temperature. The observed N-induced suppression of the surface recombination is concluded to be a result of an N-induced modification of the surface states that are responsible for the nonradiative recombination. Our results, therefore, demonstrate the great potential of incorporating GaNAs in III-V NWs to achieve efficient nano-scale light emitters.

Dilute nitride semiconductors, such as Ga(In)NAs and Ga(In)NP alloys, have been a subject of intense research efforts^1^ due to their high potential for applications in advanced optoelectronic and photonic devices such as solar cells, light-emitting-diodes (LEDs) and lasers, which are of significance in the fields of photovoltaics, medical diagnostics and fiber-optic communication. Ga(In)NAs has further been demonstrated to exhibit extraordinary spin functionalities at room temperature^2^,^3^,^4^. Recent progress in the material growth have paved the way in tailoring these materials and related heterostructures into the one-dimensional (1D) nanowire (NW) architecture, which renders the opportunity to engineer and manipulate the material properties at the nanoscale^5^,^6^,^7^,^8^,^9^,^10^,^11^,^12^,^13^,^14^,^15^,^16^. For example, the radial core/shell or core/multi shell NW design allows one to improve charge separation in NW-based solar cells. NWs can also be utilized as nanocavities with efficient confinement of both carriers and photons, as well as polarized light emitters, indispensable in photonic devices^5^,^8^,^9^,^10^,^11^,^13^,^14^. In the case of GaNP NWs, the presence of nitrogen is advantageous for further extending device functionality as it allows to achieve orthogonal polarization of the emitted light from zinc-blende (ZB) nanowires of various diameters^15^ and also leads to efficient energy upconversion via defect states^16^. On the other hand, by incorporating nitrogen in Ga(In)As, the emission energy can be tuned within the spectral window of 1.3–1.55 μm for fiber-optic communications, as a result of the giant bowing in the bandgap energy that is characteristic for dilute nitrides^1^. Moreover, the ability to grow high quality III-V NWs on foreign substrates such as Si paves the way for integration of III-V NW-based photonic devices (including those made from dilute nitrides) with the mature microelectronic technology based on Si.

An important material parameter that affects performance of the NW-based devices is carrier lifetime. In solar cells it limits a carrier diffusion length whereas in light-emitting devices it governs radiative efficiency and threshold current in laser structures. The carrier lifetime is known to be controlled by combined contributions of radiative and non-radiative recombination (NRR) processes characterized by their corresponding lifetimes. The radiative lifetime reflects efficiency of radiative recombination in the material and is determined by the oscillator strength of the corresponding optical transitions. On the other hand, the non-radiative lifetime is largely affected by the material quality both in bulk and within near-surface regions. The contribution of the surface-related recombination is known to be especially severe in GaAs-based NW structures due to a large surface-to-volume ratio and the presence of surface states participating in the NRR processes. Here, passivation of the surface states either by growing a radial shell layer of Al_x_Ga_1-x_As with a wider bandgap or by chemical treatments^17^,^18^,^19^,^20^,^21^,^22^ is of crucial importance in improving carrier lifetime.

As to GaNAs-based nanowires, detailed information regarding NRR processes is currently lacking. According to the previous studies of planar dilute nitrides, incorporation of nitrogen leads to degradation of bulk carrier lifetime caused by enhanced NRR via defects^23^,^24^,^25^,^26^. In the case of GaNP nanowires, nitrogen incorporation has also been shown to cause formation of surface defects acting as efficient NRR centers^27^,^28^. However, the opposite trend was recently reported^29^ for GaNAs-containing NWs fabricated using molecular beam epitaxy on Si substrates^30^,^31^, where room-temperature carrier lifetime was found to be improved in N-containing nanowires. This raises prospects of this NWs system for optoelectonic and photonic applications, and calls for a better understanding of the observed N-induced effect such that it can be further optimized and controlled. The purpose of this work is to shed light on the physical processes that limit carrier lifetime in GaAs/GaNAs core/shell NWs from comprehensive temperature-dependent studies of exciton and carrier dynamics based on time-resolved photoluminescence (PL) spectroscopy. Impact of the 1D architecture on the material quality will also be evaluated from comparative studies of exciton dynamics in GaNAs epilayer structures with a similar N content.

## Results and Discussion

**Figure 1:**
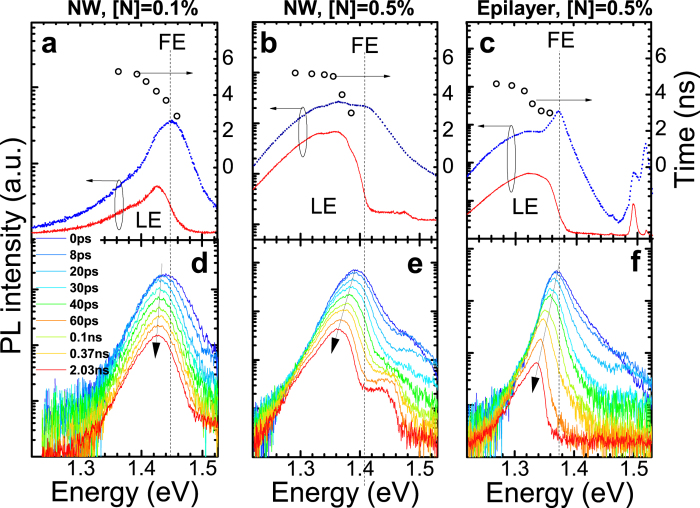
(**a**)–(**c**) PL spectra of the investigated structures measured at 5 K with the excitation power levels of 4 mW (the solid curves, red) and 55 mW (the dashed curves, blue), respectively. The open circles represent the measured PL lifetimes as a function of emission energy, with the excitation power of 55 mW. (**d**)–(**f**) PL spectra of the investigated structures detected at different time delays after an excitation pulse. The arrows indicate the shifts of the peak positions of the LE emission. The vertical dashed lines mark the FE spectral positions.

Shown as the solid curves in Fig.1a, b are PL spectra of the GaAs/GaNAs core/shell NWs with [N] = 0.1 and 0.5%, respectively, measured at 5 K with the excitation power (W_exc_) of 4 mW (focused to a spot of about 0.5 mm in diameter). For comparison, Fig. 1c also shows the PL spectrum from the GaN_0.005_As_0.995_ epilayer measured under the identical excitation conditions (the solid curve). In all structures, the spectra are dominated by a broad asymmetric PL band which originates from localized exciton (LE) recombination within the GaNAs region^29^. This emission mechanism is common for dilute nitrides where alloy disorder leads to strong fluctuations in the conduction band edge enhanced by the giant bandgap bowing effect^1^. In principle, structural polytypism in NW structures can further contribute to exciton localization, as was observed in GaAs NWs where excitons could be localized at interfaces between ZB and wurtzite (WZ) phase segments with a type-II band alignment^32^,^33^,^34^. This effect, however, does not seem to have the dominant contribution in the GaNAs NWs studied here, judging from the very similar properties of the LE emission between the NWs and epilayer structures. We should also note that the exact band alignment between ZB and WZ GaNAs is currently unknown. In addition to the LE band, the PL spectra from the GaAs/GaN_0.005_As_0.995_ core/shell NWs contain a much weaker ‘plateau’-like emission band within the 1.41–1.45 eV spectral range which stems from excitonic transition within the GaAs core region^29^. Since this weak emission only has a minor contribution in the PL spectra, it will not be further discussed in this paper. (In the case of the epilayer structure, the high-energy PL lines at 1.493 and 1.515 eV are related to free-to-acceptor (e, A^0^) and free exciton (FE) transitions from the GaAs substrate, respectively^35^). Increasing excitation power, e.g. to 55 mW, leads to a saturation of the LE states. Under these conditions, the PL spectra (shown by the dotted curves in Fig. 1a,b) contain an additional PL component that is located above the high energy cut-off of the LE band and is caused by the FE emission in GaNAs. The same tendency is also observed in the reference GaNAs epilayer (see the dotted curve in Fig. 1c). We note that the FE emission is somewhat broadened in the NW samples. This broadening is likely due to minor variations in the N composition between different NWs forming the studied NW arrays, within 0.07% for the structures with [N] = 0.5%, as was revealed by our previous micro-PL measurements^29^. The spectral positions of the GaNAs-related FE emission in the investigated structures are indicated in Fig. 1 by the vertical dotted lines.

It is well established that recombination dynamics of excitonic transitions in semiconductors reflects energy relaxation and recombination processes of non-equilibrium carriers. Therefore, in order to evaluate these processes in the GaAs/GaNAs NWs we have performed time-resolved PL measurements. Time-resolved PL spectra of the investigated structures measured at 5 K as a function of a time delay (∆t_d_) after an excitation laser pulse are shown in Fig. 1d–f. First of all, one notices that within very short time delays ∆t_d_ < 30 ps, the PL spectrum from each structure peaks at an energy close to the FE position. They are exponentially broadened at the high energy side with the PL tail extending above the FE position. This indicates a significant contribution of the FE component in the overall PL spectra. The slope of the high energy tail of the FE reflects a population distribution among the FE states determined by the exciton temperature. At later times, the PL emission is dominated by the LE transitions and undergoes a spectral red shift as marked by the arrows in Fig. 1d–f.

### Localized exciton dynamics

**Figure 2:**
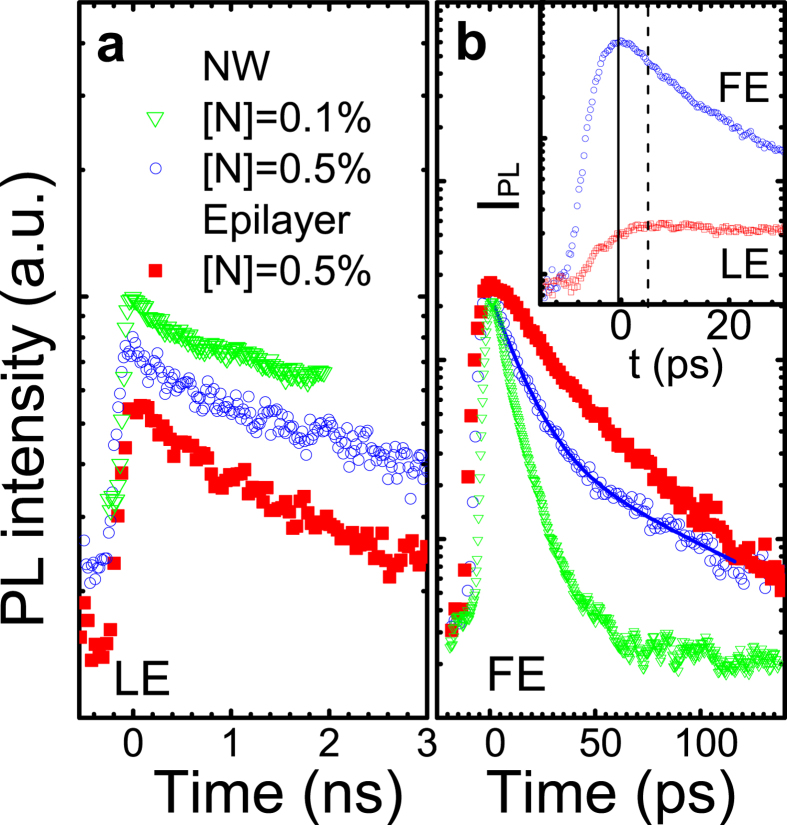
(**a**) Temporal profiles of the LE emission measured at E_det_=1.35 eV from the GaAs/GaNAs core/shell NWs with [N]=0.1% (the open triangles, green), at E_det_ = 1.29 eV from the GaAs/GaN_0.005_As_0.995_ core/shell NWs (the open circles, blue) and at E_det_ = 1.27 eV from the GaN_0.005_As_0.995_ epilayer structure (the filled squares, red). (**b**) FE decays detected at the FE peak positions from the NWs (the open symbols) and epilayer (the filled squares) structures with [N] = 0.5%. The solid line is the fitting curve assuming a bi-exponential decay function given by equation (1) with the fitting parameters as specified in the text. The inset in (**b**) gives a close-up of the FE and LE temporal profiles in the GaAs/GaN_0.005_As_0.995_ core/shell NW structure. The temporal positions of the FE and LE maximum intensity are marked by the solid and dashed line, respectively.

Let us now discuss transient properties of the LE and FE transitions in more detail. We will start this discussion with the LE emission. Consistent with the previous studies of planar dilute nitrides^36^,^37^,^38^, the LE decays are found to be predominantly single-exponential and accelerate with increasing detection energies (E_det_). This results in an apparent red shift of the LE maximum position with increasing ∆t_d_ and reflects energy relaxation from the shallow localized states to the deeper ones. The deduced decay times (τ_LE_) of the LE transitions are plotted as the open circles in Fig. 1a–c. In all structures, the energy transfer between the localized states does not contributes to the dynamics of the deeply localized excitons as τ_LE_ does not depend on E_det_ at the low energy side of the LE band. Under these conditions, τ_LE_ saturates at around 5.2 ns in the GaAs/GaNAs NW structures, which is similar to the saturation value of 4.6 ns in the reference GaN_0.005_As_0.995_ epilayer. This can also be seen from Fig. 2a where PL decays from the deeply localized states are shown. The obtained values are comparable to the radiative lifetime in bulk GaAs^39^, which implies that recombination of the deep LE is predominantly radiative, owing to localization of the exciton wavefunction within the limited volume of the GaNAs alloy which reduces probability of exciton trapping by NRR centers.

It is interesting to note that the saturation value τ_LE_ (and, therefore, the radiative lifetime τ_r_) remains constant within the temperature range of T = 4–100 K, i.e. at temperatures when the localization effects remain important. Temperature dependence of the radiative lifetime is known to be determined by dimensionality of semiconductor structures^39^,^40^,^41^. For example, it increases as 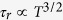 in bulk semiconductors as a result of thermally induced exciton redistribution in momentum (*k*) space. On the other hand, the temperature-independent radiative lifetime is characteristic^41^,^42^,^43^ for excitonic transitions within zero-dimensional (0D) structures, where no thermal redistribution of excitons in the *k* ~ space is expected. Assuming that the LE recombination at T < 100 K remains being governed by the radiative recombination, this suggests that the deeply localized states may experience three-dimensional quantum confinement. This conclusion is consistent with our recent results of micro-PL measurements performed on individual GaAs/GaNAs nanowires which show presence of sharp QD-like PL lines within the LE contour^29^.

### Free exciton/carrier dynamics

As is obvious from Fig. 1d–f, the FE emission at 5 K exhibits a significantly shorter decay time than the LE transitions and contributes to the PL spectra only within the first 100 ps after the laser pulse. The FE transient profiles from the investigated structures are shown by the symbols in Fig. 2(b). The FE decays can be fitted by a bi-exponential function of the form


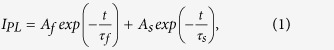


where *A*_*f*_ (*A*_*s*_) and *τ*_*f*_ (*τ*_*s*_) are the amplitude and the decay time constant of the fast (slow) decay component, respectively. In the case of the GaAs/GaN_0.005_As_0.995_ core/shell NWs, the best fit (shown by the solid line in Fig. 2b) to the experimental data (the open circles) yields *τ*_*f*_ = 13 ps, *τ*_*s*_ = 75 ps and 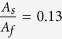. The contribution of the fast component is higher in the GaAs/GaN_0.001_As_0.999_ core/shell NWs, where *τ*_*f*_ = 9 ps. The bi-exponential decay of the excitonic emission usually implies that the monitored transitions occur in different spatial regions of the NWs with distinctly different lifetimes, e.g. in bulk and near-surface regions of the NWs. Because of a large amount of surface-related electronic states acting as efficient NRR centers, the PL lifetime (τ) in the near-surface regions is usually rather fast, as it is determined by combined contributions from the radiative and nonradiative (τ_nr_) lifetimes as described by 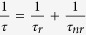. Therefore, we attribute the fast (slow) decay component to the FE recombination within the near-surface (volume) region of the GaNAs shell. This assignment is also corroborated by the observed transient behavior of the FE emission in the reference GaN_0.005_As_0.995_ epilayer (shown by the filled squares, red in Fig. 2b) where the fast decay component is largely suppressed and the exciton decay is governed by *τ*_*s*_ = 70 ps. The determined FE lifetimes are substantially shorter than that for the LE emission, which indicates an enhanced contribution of NRR in the FE decay. This is probably not surprising taking into account that the free excitons are mobile in the lattice and, therefore, have a higher chance to encounter non-radiative recombination centers. We note that at low temperatures, the determined FE lifetimes are further shortened by energy transfer to localized excitons. This process becomes apparent in temporal profiles of the FE and LE emissions measured at short time delays, as shown in the inset in Fig. 2b taking as an example the GaAs/GaN_0.005_As_0.995_ core/shell NW structure. It is obvious that the initial decay of the FE emission within the first 6 ps is accompanied by the rising of the LE emission, indicative for fast trapping of the FE excitons by the localized states.

**Figure 3:**
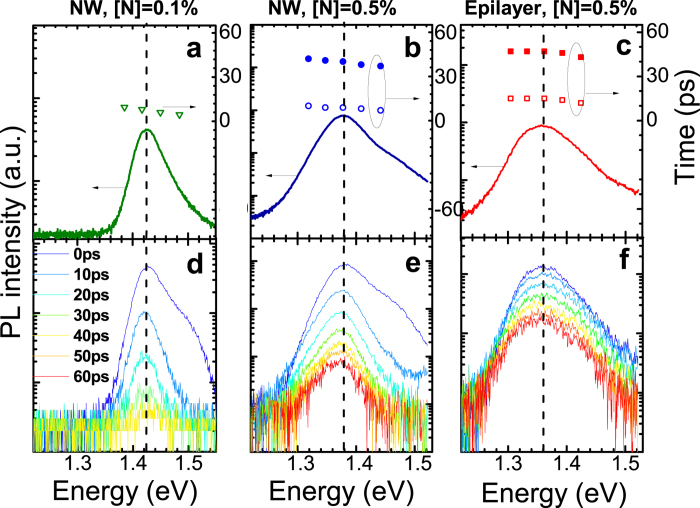
(**a**)–(**c**) PL spectra of the investigated structures measured at 140 K. The open (filled) symbols represent the deduced fast (slow) PL lifetimes as a function of emission energy. (**d**)–(**f**) PL spectra of the investigated structures detected at different time delays after the excitation pulse. The vertical dashed lines mark the FE spectral positions.

**Figure 4:**
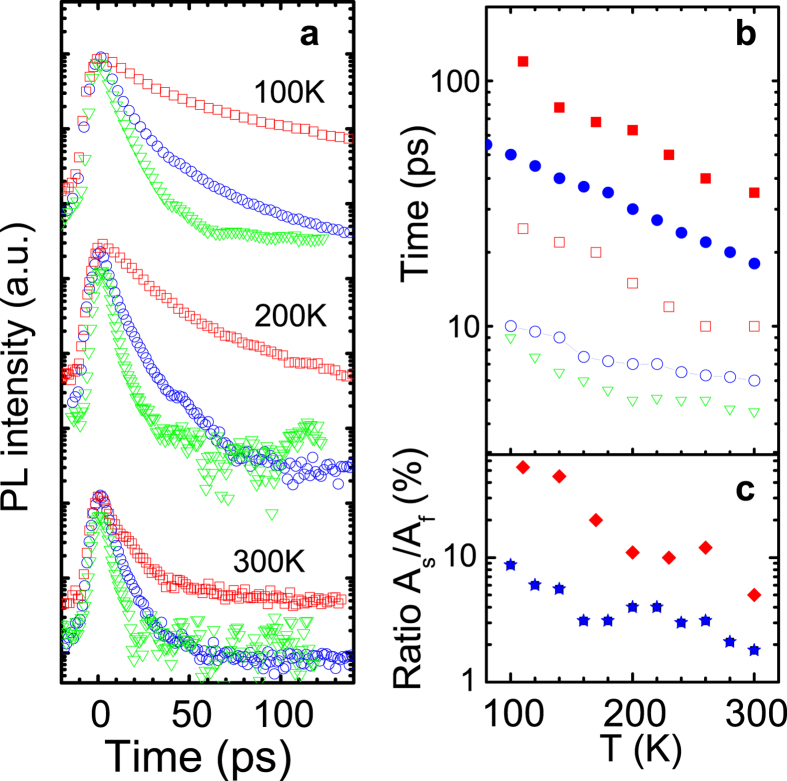
(**a**) Transient profiles of the FE/free carrier emission measured from the investigated structures at the specified temperatures. The measured decays were fitted by the bi-exponential function given by equation (1) and the deduced fast and slow time constants are shown in (**b**) by the open and filled symbols, respectively. In (**a**) and (**b**), the triangles (green), the circles (blue) and the squares (red) are the results from the GaAs/GaN_0.001_As_0.999_ core/shell NWs, GaAs/GaN_0.005_As_0.995_ core/shell NWs and the GaN_0.005_As_0.995_ epilayer, respectively. The determined *A*_*s*_/*A*_*f*_ ratio is displayed in (**c**) for the NW (stars, blue) and the epilayer (diamonds, red) structures with [N] = 0.5%. For the NW structure with [N] = 0.1%, *A*_*s*_ was set to zero.

Influence of the exciton trapping on the FE dynamics is expected to become less significant at elevated temperatures. Indeed, according to our previous studies^29^, a temperature increase causes thermal depopulation of the localized states which leads to prevalence of radiative recombination of free excitons/carriers at T > 100 K. Under these conditions, the PL spectra no longer exhibit a red shift with increasing ∆t_d_, as can be seen from Fig. 3 which shows time-resolved PL spectra (the solid curves) and decay times (the symbols) from the investigated structures at 140 K. Similar to that at 5 K, the PL decays remain bi-exponential. They accelerate with increasing T, see also Fig. 4 a where representative PL decays of the FE/free carrier emission at 100 K, 200 K and 300 K are shown. The observed reduction of carrier lifetimes with rising T reflects thermal activation of competing NRR processes degrading the PL efficiency. Simultaneously, the fast PL decay component gains its intensity and becomes dominant at room temperature not only in the core/shell NWs but also in the epilayer structure. Temperature dependences of the deduced lifetimes and the *A*_*s*_/*A*_*f*_ ratio are shown in Fig. 4b,c, respectively.

The results of the FE/free carrier dynamics presented above provide further information regarding recombination processes and material quality of the studied GaAs/GaNAs core/shell nanowires. First of all, it is obvious from Fig. 4b that the lifetimes of the slow (the filled symbols) and fast (the open symbols) components of the FE/free carrier emission are shorter in the GaNAs shell layer (the circles) than that in the reference GaNAs epilayer with the same nitrogen content (the squares). The observed shortening of the lifetimes in the NWs may indicate a stronger contribution of both volume and surface-related NRR processes, possibly due to a larger number of structural defects in the NWs caused by structural polytypism, i.e. inclusions of zinc blende and wurtzite phases, as revealed by the performed transmission electron microscopy (TEM) measurements^30^. We should note, however, that the shorter *τ*_*s*_ value in the NWs may not be solely determined by the volume quality of the GaNAs alloy. Indeed, the thickness of the GaNAs shell layer is about 150 nm, which is significantly lower than the thickness of the reference epilayer (~1 μm). As a result, volume recombination in the NW shell occurs relatively close to the surface (and also to the interface with the GaAs core) that may also shorten the measured *τ*_*s*_.

Secondly, from Fig. 2b and the temperature dependent plots of the *A*_*s*_/*A*_*f*_ ratio shown in Fig. 4c, it is obvious that though surface recombination readily plays an important role in the FE/free carrier dynamics at low temperatures its contribution significantly increases with raising T. We attribute this effect to the T-induced decrease in the carrier diffusion length caused by shortening of the carrier lifetime at elevated temperatures. Indeed, taking into account that the alloying with nitrogen does not substantially affect an absorption coefficient (α) for energies exceeding 2.2 eV^44^ and using the tabulated values of α^45^, the penetration depth of the excitation light in our experiments can be estimated as being 15 nm. Therefore, excitation of the GaNAs volume regions occurs as a result of carrier diffusion governed initially by *τ*_*f*_ and then by *τ*_*s*_ when the photo-generated carriers reach the volume region. Since both of these time constants decrease at elevated temperatures, the photo-excited region of the structure becomes restricted to the near-surface region where the surface recombination dominates.

And finally, a comparison of the PL dynamics in the core/shell NWs with different N compositions shows that effects of surface recombination become less severe with increasing N composition in the GaNAs shell. Indeed, the FE/free carrier decay time in the GaN_0.001_As_0.999_ shell is very fast, i.e. of the order of 9 ps at 100 K and further accelerates with increasing T – see Fig. 4. As a result, the majority of the photo-generated carriers recombine within the near-surface region, which degrades the overall emission efficiency. On the other hand, the decay times are longer in the GaAs/GaN_0.005_As_0.995_ core/shell NWs (see Figs 2b and 4). Consequently, it is reasonable to expect that the contribution of the volume-related recombination is more pronounced in this structure, as the photo-excited excitons/free carriers no longer solely reside within the near-surface region. This is indeed observed experimentally, obvious from the appearance of the slow decay component in the PL transients – see Figs. 2b and 4. The observed slow-down of the surface recombination rate leads to a substantial (by about 20 times) increase in the PL intensity when the N composition in the shell changes from 0.1% to 0.5%. A possible reason of the observed suppression of the surface recombination could be partial nitridation of the GaNAs surface during the growth. Previous studies of GaAs surfaces^46^ and NWs^22^ have established that chemical nitridation of the GaAs surface in hydrazine sulfide solution substantially reduces surface defect density and, consequently, surface recombination velocity. This effect is attributed to the formation of stable Ga-N bonds at the GaAs surface which causes passivation of Ga dangling bonds and inhibits surface oxidation. We speculate that to a certain extent the same process may happen *in-situ* during the growth of the GaNAs shell, facilitated by the supply of N atoms from a N plasma. Indeed, the nanowire growth was carried out under As and N overpressure conditions. At the end of the nanowire growth, the flux of group III Ga was steeply terminated by the shutter control. On the other hand, the atmospheric As and residual N were kept supplied to the nanowires, as these processes cannot be completely controlled by a shutter^47^,^48^. In contrast to the desorption of As, the active nitrogen adsorbs on the surface with unit sticking efficiency^49^. Consequently, the residual N would substitute the topmost As, providing an increased number of Ga-N bonds at the nanowire surface and, therefore, its supplementary nitridation. This is also consistent with our results from micro-Raman measurements which shows that the total number of Ga-N bonds in the NW structures exceeds what is expected for a given alloy composition. Such a reduction in surface recombination could, therefore, be of importance for improving efficiency of radiative recombination in GaNAs-based nanostructures.

### Summary

In summary, we have utilized time-resolved PL spectroscopy to study exciton and free carrier recombination dynamics in GaAs/GaNAs core/shell NWs. It is shown that the PL emission in these structures is dominated by radiative recombination of localized excitons at T < 100 K and free exciton/carriers at T > 100 K, both of which occur within the GaNAs shell. The LE dynamics is found to be determined by their energy transfer from shallow to deep localized states and also radiative recombination. The latter has a typical lifetime of around 5 ns and predominantly governs transient behavior of the deeply localized excitons. The fact that this radiative lifetime is insensitive to measurement temperature could suggest that the deeply localized states within the GaNAs alloy experience three-dimensional quantum confinement. On the other hand, PL decay arising from FE/free carriers that dominates at T > 100 K is found to be significantly faster and is governed by nonradiative recombination which occurs predominantly within the near-surface region of the GaNAs shell. The contribution of the surface recombination, which shortens the exciton lifetime down to tens of picoseconds, is concluded to be enhanced in the NW structures with a higher surface-to-volume ratio. This conclusion is based on the comparison of exciton dynamics in the NW structures with that in the reference GaNAs epilayer. Most importantly, the surface recombination becomes partially suppressed with increasing nitrogen composition in the alloy. This is attributed to an N-induced modification of the surface states that are responsible for non-radiative carrier recombination. Our results, therefore, demonstrate the great potential of incorporating GaNAs in III-V NWs to achieve efficient nano-scale light emitters, which offers a number of attractive added values such as large freedom in band and lattice engineering as well as inherent passivation of harmful surface states.

## Samples and Methods

The investigated uncapped GaAs/GaNAs core/shell NWs were grown by plasma-assisted molecular beam epitaxy (MBE) on (111) plane Si substrates using as a catalyst Ga droplets formed on the substrate surface. The axial growth of the GaAs core was performed at 570 ^o^C via the vapor-liquid-solid (VLS) mechanism. The GaNAs shell was then grown at a lower growth temperature (T_g_) of 430 ^o^C with a N plasma ignited via step-mediated growth. The desired N compositions of [N] = 0.1% and 0.5% were achieved by controlling the N flux. The lateral growth rate, namely the rate of the wire shell grown under the vapor-solid mode, was found to be slower by about 20% as compared with that of the planar thin film, probably due to a different arriving rate of adatoms to the growth front. On the other hand, the rate can be controlled linearly by the growth time and the flux of group-III atoms, identical to that for the standard thin films^31^. This enables the accurate control of the wire diameter and compositions of the compound layers. The nitrogen composition within the GaNAs shell layers was slightly higher (by up to 0.1% ) as compared with that in simultaneously grown thin films^31^. This difference likely stems from the different arriving rate between the elemental group III atoms and active plasma species of N. Overall, the growth mechanism of GaNAs including the solubility of N in the wire shell seems to be similar to that for the planar thin films. The continuous supply of the N plasma flux to the growing wire is expected to result in its homogeneous distribution within the shell. Based on the performed scanning electron microcopy (SEM) measurements, the nanowires have a hexagonal cross-section and a total diameter of 350–400 nm, and are 3–4.5 μm long. The diameter of the GaAs core within the NW structure is about 150 nm. According to the performed TEM measurements^30^, the NWs have predominantly ZB structure. A detailed description of the growth process can be found in Ref. 30 and 31. To compare material quality of the GaNAs alloy in the core/shell NWs with that in planar structures, we have also studied a GaN_0.005_As_0.995_ epilayer grown by MBE on a semi-insulating (100) GaAs substrate.

Time-resolved PL measurements were performed over the temperature range of 4–300 K in a temperature variable cryostat. A wavelength tunable Ti: Sapphire pulsed laser set at 380 nm was used as an excitation source, with a repetition rate of the laser pulses at 76 MHz and spectral and temporal width of 1 nm and 2 ps, respectively. This excitation wavelength was chosen to selectively probe effects of surface recombination. The PL transients were detected by a streak camera assembled with a single grating monochromator. Excitation power dependent PL measurements were done using the same laser operating in a continuous wave (cw) mode. The cw – PL signal was spectrally dispersed by a 0.5 m single grating monochromator and recorded by a charge coupled device (CCD).

## Additional Information

**How to cite this article**: Chen, S. L. *et al.* Suppression of non-radiative surface recombination by N incorporation in GaAs/GaNAs core/shell nanowires. *Sci. Rep.* **5**, 11653; doi: 10.1038/srep11653 (2015).
